# Transplantation of adipose tissue-derived microvascular fragments promotes therapy of critical limb ischemia

**DOI:** 10.1186/s40824-023-00395-6

**Published:** 2023-07-16

**Authors:** Gyu Tae Park, Jae Kyung Lim, Eun-Bae Choi, Mi-Ju Lim, Bo-Young Yun, Dae Kyoung Kim, Jung Won Yoon, Yoon Gi Hong, Jae Hoon Chang, Seong Hwan Bae, Jung Yong Ahn, Jae Ho Kim

**Affiliations:** 1 Department of Physiology, College of Medicine, Pusan National University, Yangsan, Gyeongsangnam-do 50612 Republic of Korea; 2 UVA Surgery Clinic, Busan, 47537 Republic of Korea; 3 BS The Body Aesthetic Plastic Surgery Clinic, Busan, 47287 Republic of Korea; 4 Department of Plastic and Reconstructive Surgery, College of Medicine, Pusan National University, Busan, Gyeongsangnam-do 49241 Republic of Korea; 5 Department of Physiology, Pusan National University School of Medicine, Yangsan, Gyeongsangnam-do 50612 Republic of Korea

**Keywords:** Mesenchymal stem cells, Microvascular fraction, Endothelial progenitor cells, Peripheral artery diseases, Therapeutic angiogenesis

## Abstract

**Background:**

Adipose tissue-derived microvascular fragments are functional vessel segments derived from arterioles, capillaries, and veins. Microvascular fragments can be used as vascularization units in regenerative medicine and tissue engineering containing microvascular networks. However, the in vivo therapeutic and vascularization properties of human microvascular fragments have not been investigated.

**Methods:**

In this study, we isolated microvascular fragments, stromal vascular fractions, and mesenchymal stem cells from human lipoaspirate and studied their therapeutic efficacy and in vivo vasculogenic activity in a murine model of hindlimb ischemia. In addition, in vivo angiogenic activity and engraftment of microvascular fragments into blood vessels were measured using Matrigel plug assay.

**Results:**

Both microvascular fragments and stromal vascular fractions contain not only mesenchymal stem cells but also endothelial progenitor cells. In a Matrigel plug assay, microvascular fragments increased the number of blood vessels containing red blood cells more than mesenchymal stem cells and stromal vascular fractions did. The engraftment of the microvascular fragments transplanted in blood vessels within the Matrigel plug significantly increased compared to the engraftment of mesenchymal stem cells and stromal vascular fractions. Moreover, intramuscular injection of microvascular fragments markedly increased blood flow in the ischemic hindlimbs and alleviated tissue necrosis compared to that of mesenchymal stem cells or stromal vascular fractions. Furthermore, transplanted microvascular fragments formed new blood vessels in ischemic limbs.

**Conclusions:**

These results suggest that microvascular fragments show improved engraftment efficiency and vasculogenic activity in vivo and are highly useful for treating ischemic diseases and in tissue engineering.

**Graphical Abstract:**

Adipose tissue-derived microvascular fragments are vascularization units in regenerative medicine and tissue engineering containing microvascular networks. Intramuscular injection of microvascular fragments markedly increased blood flow in the ischemic hindlimbs and alleviated tissue necrosis. The present study suggests that microvascular fragments show improved engraftment efficiency and vasculogenic activity in vivo and are highly useful for treating ischemic diseases and in tissue engineering.

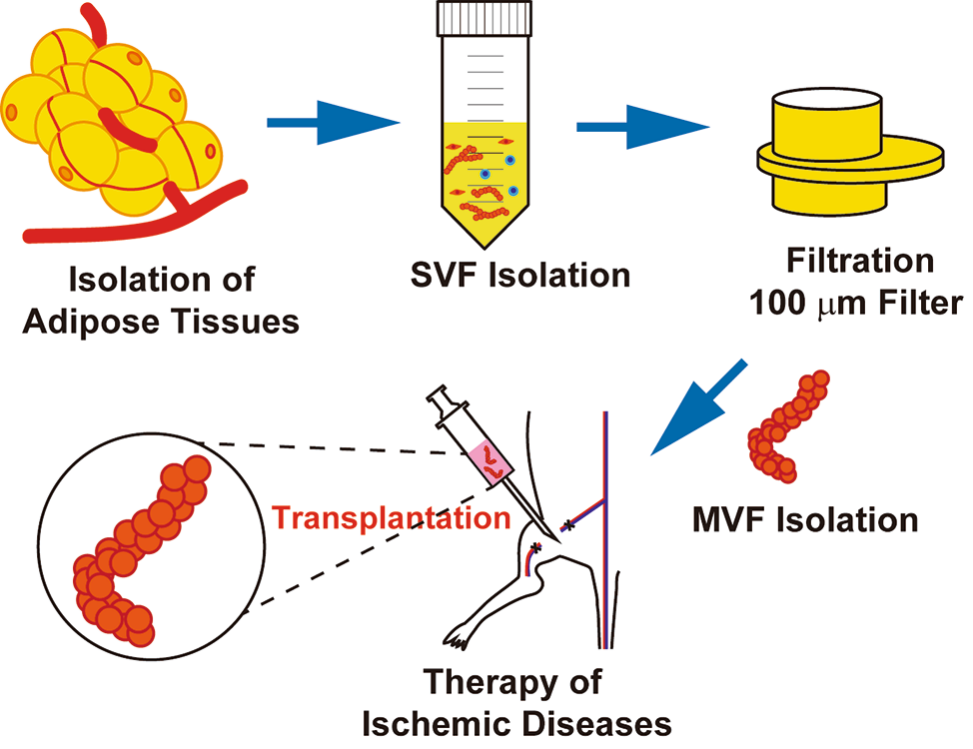

**Supplementary Information:**

The online version contains supplementary material available at 10.1186/s40824-023-00395-6.

## Introduction

Peripheral artery disease (PAD) is a cardiovascular disease caused by the occlusion of peripheral arteries [1]. It is characterized by obstruction of the blood vessels in the lower extremities, caused by narrowing of the arteries due to the buildup of plaques composed of excess cholesterol, fatty substrates, and blood clots in the vessel walls [2]. PAD is associated with diabetes, atherosclerosis, and other cardiovascular diseases, and it causes tissue necrosis in peripheral areas, such as the feet and legs [3, 4]. Patients with severe PAD can be treated with either vascular bypass surgery or endovascular ste-nt placement to expand the narrowed blood vessels [5–7]. However, these treatments have critical limitations that result in temporary side effects caused by the implants. Therapeutic angiogenesis, including stem cell therapy, has drawn attention as a potential novel therapy for PAD [8–10].

Adipose tissue is a multifunctional organ that contains multiple cell types, including stromal vascular fractions (SVFs) and mature adipocytes. The SVFs are a mixture of various cell types, including mesenchymal stem cells (MSCs), endothelial cells, fibroblasts, monocytes, and macrophages [11, 12]. SVFs can be isolated by enzymatic digestion of adipose tissue and centrifugal separation. By culturing SVFs on culture dishes, colony-forming cells can be isolated and amplified to form adipose stromal cells or MSCs upon further cell culture [13]. MSCs are promising candidates for tissue engineering and cell therapy because of their multipotent differentiation capability and paracrine function [14]. MSCs secrete various paracrine factors, including growth factors and proangiogenic cytokines, which play key roles in treating PAD [15]. MSC-derived cytokines and paracrine factors, such as IL-6, IL-8, IL-10 and VEGF, have been shown to stimulate angiogenesis and tissue regeneration in ischemic tissues [16–18]. Not only MSCs but also SVFs has been reported to restore the blood flow in a rat with critical limb ischemia [19]. Despite the clinical usefulness of the MSCs and SVFs, their therapeutic efficacy for ischemic diseases has been hampered by the poor engraftment of transplanted cells [20]. Reduced oxygen supply and high levels of inflammatory cytokines in ischemic tissues cause the death of transplanted cells [21]. The transplanted MSCs exhibit poor ability to differentiate into vascular smooth muscle cells in vivo [22, 23], and the in vivo survival rate and engraftment ability of transplanted SVFs are quite low [24, 25]. Therefore, it is necessary to improve the engraftment and survival rates of the transplanted MSCs and SVFs.

Microvascular fragments (MVFs) are small functional vascular units that can be isolated from the adipose tissue [26]. Longer enzymatic digestion of adipose tissue mainly yields SVFs composed of various single cells [27, 28], whereas shorter digestion yields a mixture of single cells and adipose tissue-derived MVFs [29]. These MVFs represent intact vessel segments and, thus, exhibit the unique feature of rapidly reassembling into new microvascular networks after transplantation [26]. MVFs demonstrate significantly higher viability and vasculogenic ability in vivo than those of SVFs [30]. Co-transplantation with MVFs promotes angiogenesis and revascularization of transplanted dental pulp stem cell aggregates, leading to robust dental pulp regeneration [31]. Moreover, MVF-seeded skin grafts improve implant integration within the host tissue in a full-thickness wound model by promoting the formation of microvascular networks and interconnection with the host microvasculature [32, 33]. However, the therapeutic efficacy and vasculogenic ability of MVFs have not yet been explored in ischemic disease models.

In this study, we isolated MSCs, SVFs, and MVFs from lipoaspirate of human donors and compared their therapeutic efficacy in an animal model of hindlimb ischemia. In addition, we measured the engraftment efficiency and blood vessel-forming ability of the transplanted cells in ischemic limbs and Matrigel plugs. We demonstrate for the first time that MVFs exhibits superior therapeutic and engraftment efficacies in vivo compared to MSCs and SVFs, suggesting that MVFs is a highly useful cell source for the regeneration therapy of ischemic diseases.

## Material & methods

### Materials

Collagenase-1, bovine serum albumin (BSA), and 2,2,2-Tribromoethanol was purchased from Sigma-Aldrich (Saint Louis, MO, USA). Nylon mesh were purchased from pluriSelect life science (Leipzig, Germany). Accutase, HBSS, Fetal Bovine Serum, and alpha MEM were purchased from Invitrogen (Carlsbad, CA, USA). Human enzyme linked-immunosorbent assay (ELISA) kits for IL-6 (430,504), IL-8 (431,504) were purchased from BioLegend (San Diego, CA, USA), VEGF(DY239B) and IL-10(DY217B) were purchased from R&D Systems (Minneapolis, MN, USA). Flow cytometry and Immunostained antibodies were listed in Table S1.

### Isolation of MVFs and mesenchymal stem cells

The MVFs were isolated from the lipoaspirate of patients undergoing liposuction surgery by using a modified protocol described in a previous report [34]. Informed consent was obtained from each donor. This study was approved by the Institutional Review Board of the Pusan National University Hospital (1801-033-062). The clinical information of the 11 patients who provided adipose tissues is summarized in Table S2. Aspirated subcutaneous adipose tissues obtained from donors undergoing plastic surgery were washed with HBSS and centrifuged at 1300×*g* for 10 min to separate fatty and fluid portions (Fig. S1). The fatty portion (upper fraction) was mixed with an equal volume of 0.1% type-1 collagenase solution and incubated at 37 °C for 40 min with constant shaking (200 rpm). The collagenase digests were subjected to centrifugation, and the pellets were resuspended in HBSS and washed by re-centrifugation. The enzyme digests were sieved through a bovine serum albumin (BSA)-coated nylon mesh, which was pre-treated with HBSS containing 5% BSA overnight to prevent the MVFs from sticking to the mesh. BSA coating of nylon mesh increased purification yield of MVFs from the enzyme digests. The undigested adipose tissue was retained in the mesh, and the SVFs passed through the mesh. The flow-through SVFs were collected and passed through a BSA-coated second mesh with different pore sizes to separate MVFs and single cells from the SVFs. The MVFs retained on the mesh were collected in ice-cold HBSS by gentle pipetting and centrifugation at 1000×*g* for 5 min. The MVFs were resuspended in PBS and counted under a microscope for MVFs longer than 100 μm in length. To isolate the MSCs from SVFs, it was resuspended in α-minimum essential medium supplemented with 10% fetal bovine serum and 100 U/mL penicillin-streptomycin, after which the cells were seeded in tissue culture dishes at a density of 3,500 cells/cm^2^. The primary MSCs were cultured for 4–5 days in an incubator set at 37 °C and 5% CO_2_ until they reached confluence; this was defined as passage “0.” The passage number of the MSCs used in our experiments was between 5 and 10. After trypsin treatment of MSCs, SVFs, and MVFs, the numbers of viable cells in these fractions were quantified by trypan blue staining with a hemocytometer.

### Flow cytometry analysis

The MVFs and SVFs were dissociated using Accutase and filtered through a 40-µm nylon mesh. The dissociated cells were incubated with antibodies against APC-conjugated antibodies (CD29, CD44, and CD45), PE-conjugated antibodies (anti-CD90, CD105, and CD117), FITC-conjugated antibodies (CD31, CD34, and CD38), and eFluor^TM^506-conjugated antibody (anti-CD14). Fluorescent antibody-conjugated cells were counted using the Attune flow cytometer.

### Immunocytochemistry

MVFs were collected through cytospin centrifugation (1500 rpm for 3 min) and mounted on silane-coated slide glass without ECM coating. Samples were then blocked with 5% BSA for 1 h and incubated with primary antibodies, such as smooth muscle cell markers (α-SMA), endothelial cell markers (vWF and isolectin B4 (ILB4)), and mesenchymal cell markers (vimentin, CD44, and CD105). The primary antibodies were incubated overnight at 4°C. The slides were washed and stained with secondary antibodies (Alexa Fluor 488, 568) at 25 °C for 2 h. The stained slides were mounted with anti-fade solutions with DAPI (4’,6-Diamidino-2-phenylindole dihydrochloride) and imaged using Zeiss confocal instruments.

### Hindlimb ischemia animal model

Six-week-old male BALB/CA-nu/nu mice (average weight: 20–24 g) were purchased from Orient Bio (Seongnam-si, Korea). All animals were bred in a humidity-controlled, air-conditioned animal room and were provided with laboratory animal feed and water. After the mice were anesthetized with an intraperitoneal injection of 400 mg/kg 2,2,2 tribromoethanol (Avertin; Sigma-Aldrich), the femoral artery segment between its proximal origin at the branch of the external iliac artery and its distal bifurcation into the saphenous and popliteal arteries was resected. After arterial resection, the same cell numbers (1 × 10^6^ cells/mouse unless otherwise indicated) of MSCs, SVFs and MVFs were injected at four sites (20 µL at each site) of the gracilis muscle in the medial portion of the thigh. MVFs and SVFs were transplanted into ischemic limbs without enzymatic dissociation into single cells. The blood flow in the hindlimb was measured on days 0, 7, 14, 21, and 28 after induction of hindlimb ischemia using a laser Doppler perfusion imaging (LDPI) analyzer (Moor Instruments Ltd., Devon, UK). The perfusion of ischemic and non-ischemic limbs was calculated based on the type of pixels generated on the color histogram. Red and blue indicate high and low perfusion levels, respectively. Hemoperfusion is expressed as the LDPI index, which represents the ratio of ischemic to non-ischemic limb blood flow. A preoperative ratio of 1 indicates equal blood perfusion in both the feet. The degree of ischemic hindlimb necrosis was recorded 28 days after surgery. The necrosis score was evaluated using the following: 0, limb recall; 1, toe amputation; 2, foot amputation; 3, limb amputation.

### Matrigel plug assay

A Matrigel plug assay was performed to evaluate the in vivo angiogenic potential of the cells. Briefly, the BALB/c nude mice were anesthetized and subcutaneously injected with 500 µL of growth factor-reduced Matrigel containing MVFs, SVFs, and MSCs (1 × 10^6^ cells/mouse). Matrigel plugs were excised after two weeks and stained with hematoxylin and eosin (H&E) to observe the formation of new blood vessels. We also determined the hemoglobin content in the Matrigel plugs using a hemoglobin assay kit. Matrigel plugs were homogenized in a water-heparin solution and centrifuged at 1,500×*g* for 15 min at 20 °C. The hemoglobin content of the supernatant (100 µL) was determined using the Drabkin’s method [35] at 540 nm using spectrophotometry. The Matrigel plug was fixed with 4% paraformaldehyde and embedded in paraffin to measure vascularization by histological analysis. The embedded specimens were stained using H&E and immunohistochemistry stains.

### Immunohistochemistry

To confirm vascularization in Matrigel plugs, slide samples were deparaffinized with xylene and blocked with M.O.M.® IgG blocking reagent (Vector Laboratories, Newark, CA, USA) for 1 h. The blocked specimens were incubated with primary antibodies. The information about the primary antibodies used in this study are listed in Table S1. To confirm angiogenesis in the hindlimb tissues, the slides were stained with immunohistochemistry stains. Briefly, fixed muscles were embedded in Tissue-Tek® O.C.T compound (Sakura Finetek USA, Inc., Torrance, CA), incubated at room temperature, and washed to remove the Tissue-Tek® O.C.T. compound. The washed samples were blocked with M. O. M. ® IgG blocking reagent for 1 h and incubated with primary antibodies (anti-α-SMA, ILB4, and HNA). The samples were then washed and incubated with secondary antibodies (Alexa Fluor 488, 568, and 647) at room temperature for 2 h. The stained samples were thereafter mounted on slides using prolonged gold anti-fade mounting solutions and visualized using Zeiss confocal instruments.

### Cell migration assay

Human endothelial progenitor cells (EPCs) were isolated from human umbilical cord blood as previously reported [36]. Cell migration of EPCs was assayed using a disposable 96-well chemotaxis chamber. EPCs were harvested with 0.05% trypsin containing 0.02% EDTA, washed once, and resuspended in EBM-2 at a concentration of 5 × 10^3^ cells/ml. A chemotaxis chamber with an 8 μm pore size membrane filter was pre-coated overnight with 20 µg/ml rat-tail collagen at 4 °C. To measure the effects of conditioned medium from MSCs (MSC-CM), SVFs (SVF-CM), and MVFs (MVF-CM) on EPC migration, an aliquot (35 µL) of the EPC suspension was loaded into the upper chamber, and the conditioned medium was then placed in the lower chamber. After incubation of the cells for 12 h at 37 °C under 5% CO2, the filters were disassembled, and the upper surface of each filter was scraped free of cells by 3 wiping with a cotton swab. The number of cells that migrated to the lower surface of each filter was determined by counting the cells in five random locations under a microscope at ×100 magnification after staining with DAPI.

### Tube formation assay

To measure the tube-forming ability of EPCs, growth factor-reduced Matrigel (BD Biosciences) was added to 96-well culture plates and polymerized for 30 min at 37 °C. An aliquot of EPCs (1.0 × 10^4^ cells) was seeded on these Matrigel-coated plates and cultured in EBM-2 medium supplemented with VEGF, MSC-CM, SVF-CM, and MVF-CM. After incubation of the cells at 37 °C under 5% CO2 for 12 h, the capillary structures were photographed with a digital camera in four random microscopic fields and quantified by measuring the capillary length using the Image J software (version 1.50i).

### Enzyme-linked immunosorbent assay (ELISA)

Commercially available sandwich ELISA kits were used to evaluate the protein levels of IL-6, IL-8, IL-10 and VEGF in the conditioned medium derived from MSCs, SVFs, and MVFs. In brief, MSCs, SVFs, and MVFs were seeded in wells of a 24-well culture plate at a density of 1 × 10^4^ cells/well and cultured for 48 h to confluence in the growth medium. After treatment of the cells with serum-free medium supplemented with appropriate reagents, conditioned medium was collected and centrifuged at 15,000 × g for 30 min to remove particulates. ELISA of the conditioned medium was carried out according to the manufacturer’s protocol. The absorbance (450 nm) for each sample was analyzed by an ELISA reader and was interpolated with a standard curve.

### Statistical analysis

The results of the multiple observations are presented as the mean ± standard deviation (SEM). Comparisons between the two groups were performed using the student’s *t*-test. For multivariate data analysis, group differences were assessed using one-way or two-way ANOVA, followed by Scheffe’s post hoc test.

## Results

### Isolation of MVFs from lipoaspirates

**Fig. 1:**
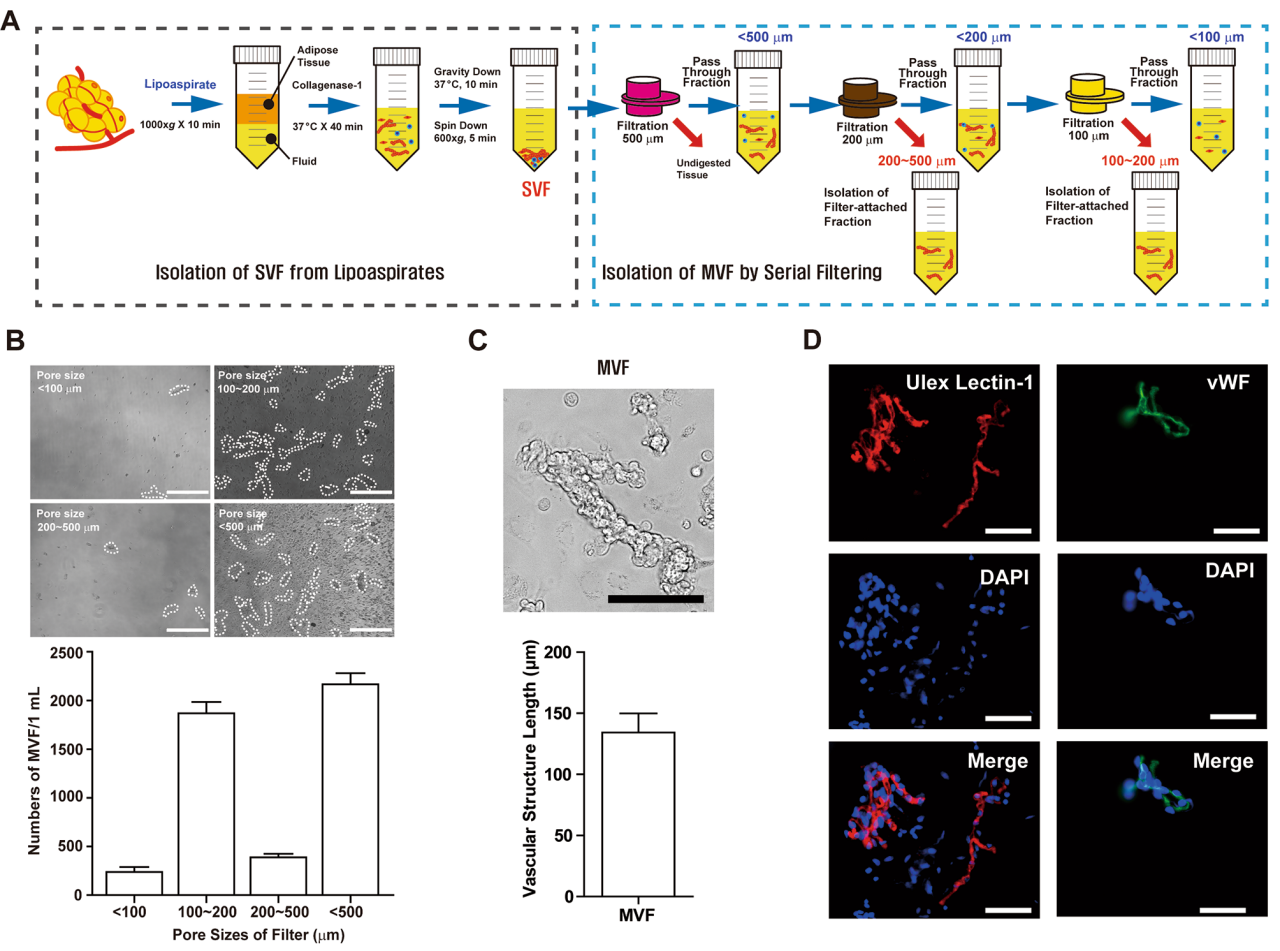
**Establishment of the protocol for MVF isolation from human adipose tissue** (**A**) Schematic representation of the protocol for MVF and SVF isolation from adipose tissues. The SVFs were serially sieved with 500-µm, 200-µm, and 100-µm pore meshes, and the pass-through fractions and the fractions attached on the meshes were collected. (**B**) The image of MVFs isolated by sieving with meshes. The MVFs were photographed under a microscope (Scale bar = 400 μm). Dashed lines indicate the MVFs (upper panel). The MVF numbers of the pass-through fractions and the fractions attached on the meshes (lower panel). *p < 0.05 by unpaired t-test (n = 3). (**C**) Representative images of MVFs (upper panel). Scale bar = 100 μm. The length of MVFs was measured under a microscope and showed as mean ± SD (lower panel). (**D**) Immunofluorescence staining of MVFs for endothelial markers. MVFs were stained with Ulex europaeus lectin-1 and anti-vWF antibody, counter-stained with DAPI, and the immunofluorescence images were overlaid. Scale bar = 50 μm

It has been reported that MVFs can be isolated from murine adipose tissue by limited digestion for ~ 10 min with collagenase and sieving with a 500 μm mesh to remove the fat clots [37]. SVFs can be isolated from the adipose tissue by prolonged enzymatic digestion for 40–60 min and centrifugation [38]. To optimize the isolation protocol of human MVFs and SVFs, the lipoaspirates were digested by treatment with type-1 collagenase at different time points, followed by measurement of the number of MVFs in the SVFs. The number of MVFs in the SVFs was maximum after treatment of the lipoaspirates with collagenase for 40 min (Fig. S1C). The collagenase-treated lipoaspirates were sieved through BSA-coated 500 μm nylon mesh to prevent the MVFs from sticking to the mesh. BSA-coating of nylon mesh increased purification yield of MVFs from the enzyme digests (Fig. S1E). To further separate the MVFs from SVFs, the SVFs were resuspended and filtered with 500 μm, 200 μm, 100 μm, and 50 μm nylon meshes (Fig. 1A). The number and length of MVFs were measured under a microscope. Most of the MVFs passed through meshes with pores of 500 μm and 200 μm size, whereas they were mostly captured with a 100 μm-pore mesh (Fig. 1B), suggesting that MVFs had a size of approximately 100 μm. Usually, 2 × 10^6^ MVFs could be isolated from 100 mL of lipoaspirate. When the length of MVFs was measured under a microscope, the MVFs exhibited a capillary-like structure with a length of 134 ± 38 μm (Fig. 1C). The MVFs could be stained with Ulex europaeus lectin-1 and anti-vWF antibody, which are markers for vascular endothelial cells (Fig. 1D). Therefore, in the following experiments, we used stepwise filtration using 500 μm pore mesh, followed by 100 μm pore mesh to remove the fat and isolate the MVFs.

### Phenotypic characterization of MVFs

**Fig. 2:**
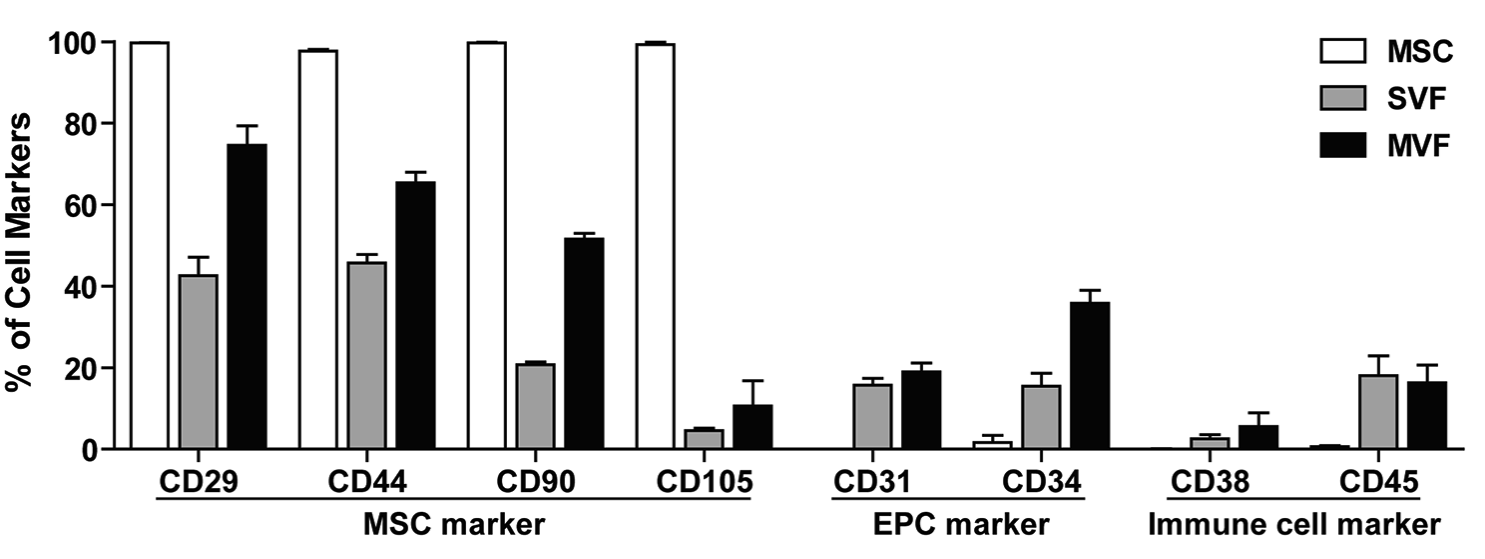
**FACS analysis of MSCs, SVFs, and MVFs**. FACS analysis of MSCs, SVFs, and MVFs with antibodies against MSC markers (CD29, CD44, CD90, CD105), EPC markers (CD31, CD34), and immune cell markers (CD38, CD45). The percentage of each cell population was quantified and the data are shown as mean ± SD (n = 3)

It has been previously reported that MVFs contain endothelial and smooth muscle cells [34]. In the present study, the phenotypes of the MVFs were characterized using immunocytochemical analysis. MVFs expressed endothelial cell markers (ILB4 and vWF), smooth muscle markers (α-SMA), and mesenchymal cell markers (CD44 and vimentin) (Fig. S2). To further confirm the presence of MSCs in MVFs, the cell components in the MVFs that had dissociated into single cells were characterized by flow cytometry. The MSCs were positive for CD29, CD44, CD90, and CD105, which are MSC markers (Fig. 2 and S3A). Both MVFs and SVFs contained CD29-, CD44-, and CD90-positive populations; however, they were not positive for CD105. Approximately 20% of MVFs were CD31-positive, and 37% were CD34-positive; these markers are expressed in the EPCs. The cell populations of MVFs expressing CD34 were greater than those of the SVFs (Fig. 2 and S3B). These results suggest that MVFs contain both MSCs and endothelial cell types. Moreover, CD38-positive lymphocytes were not detected in the MVFs, SVFs, and MSCs; however, a small population of CD45-positive hematopoietic cells could be detected in the MVFs and SVFs, but not in MSCs (Fig. 2 and S3C). The number of F4/80-positive macrophages in MVFs was significantly less than that in SVFs (Fig. S4). These results suggesting that MSCs and EPCs were more enriched in MVFs.

### Enhanced vasculogenic activity of MVFs

**Fig. 3:**
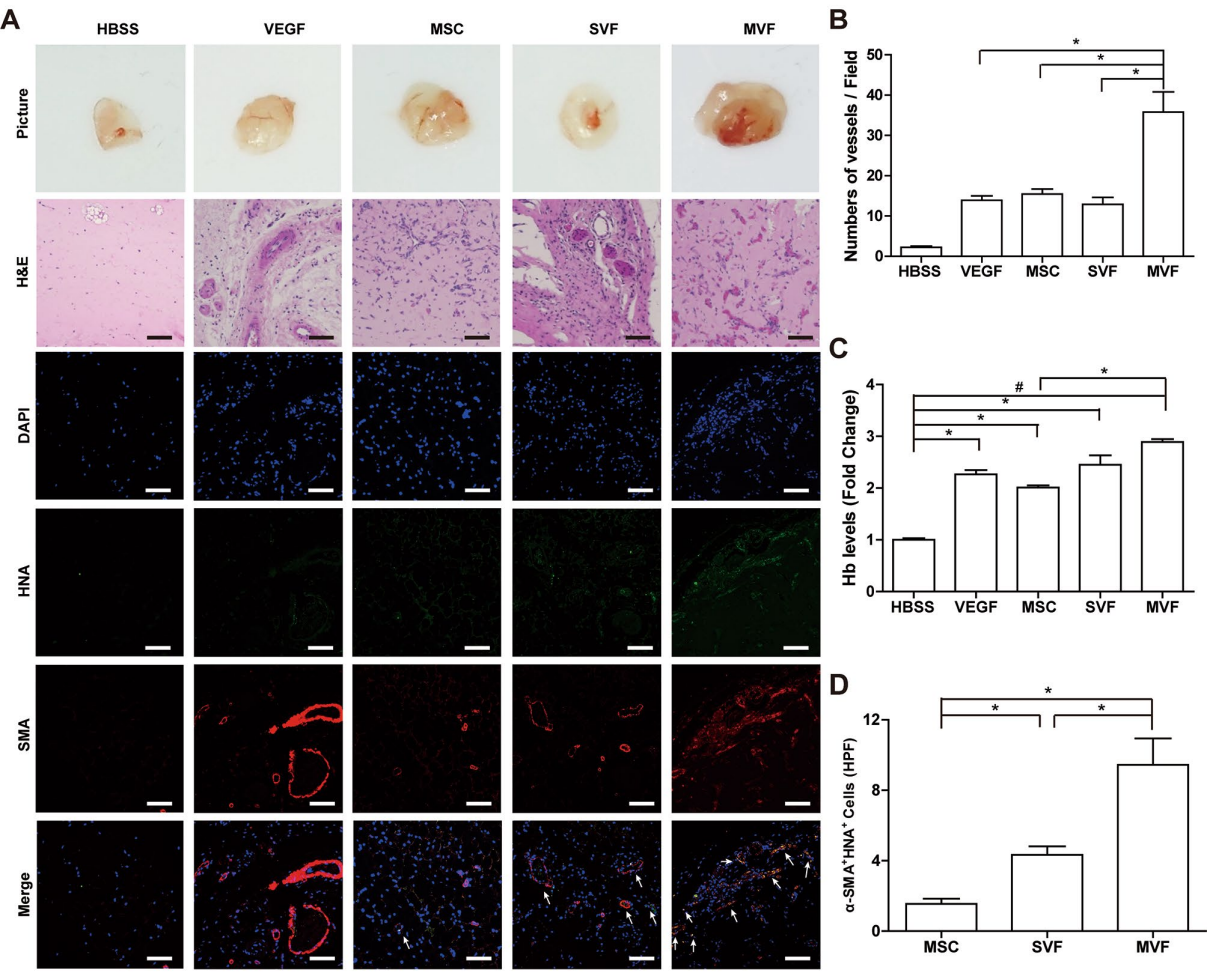
**In vivo Matrigel plug assay of MVFs, SVFs, and MSCs** (**A**) Photographs, H&E staining, and immunostaining of blood vessels of Matrigel plugs. (Top panel) Matrigel solution containing MVFs, SVFs, MSCs, and VEGF was subcutaneously injected, and the representative images of Matrigel plugs collected after 14 days are shown. (Middle panel) Histological analysis of the Matrigel plugs with H&E staining. The newly formed blood vessels containing red blood cells are indicated by arrows. (Bottom panel) Immunostaining of the blood vessels with antibodies against α-SMA and HNA. The nuclei were stained with DAPI, and overlaid images are shown. Scale bar = 100 μm. (**B**) Quantification of the number of blood vessels in the H&E-stained Matrigel plugs (n = 4). (**C**) Quantification of hemoglobin levels in the Matrigel plugs using a hemoglobin assay kit (n = 6). (**D**) Measurement of blood vessel formation by transplanted cells. The numbers of HNA- and α-SMA double positive vessels were counted (n = 4). Data indicate mean ± SD. ^#^p < 0.01, *p < 0.05

The vasculogenic activities of MVFs, SVFs, and MSCs were measured using a Matrigel plug assay. A Matrigel solution containing MVFs, SVFs, MSC, and recombinant vascular endothelial growth factor (VEGF) protein was subcutaneously injected, and the Matrigel plugs were collected for analysis of angiogenesis. The MVF-containing Matrigel plugs exhibited greater redness than those containing MSCs, SVFs, and VEGF. Blood vessel formation was analyzed using hematoxylin and eosin staining (Fig. 3A). The Matrigel plugs exhibited red blood cell-containing blood vessels, and the number of blood vessels was more significant in Matrigel plugs injected with MVFs than in those injected with MSCs and SVFs (Fig. 3B). The levels of hemoglobin in the Matrigel plugs were measured using a hemoglobin assay. Hemoglobin levels increased in all experimental groups but were higher in the MVFs group than in the other groups (Fig. 3C). To measure the vasculogenic ability of MVFs, the Matrigel plugs were stained with antibodies against α-SMA, a smooth muscle marker, and a human nuclei antigen (HNA). As shown in Fig. 3D, the number of SMA- and HNA-double-positive blood vessels in the MVF-injected Matrigel plugs was greater than that in the MSC- or SVF-injected Matrigel plugs (Fig. 3A and D). Furthermore, MVF-injected Matrigel plugs showed a significant increase in the number of CD31- and HNA-double positive capillaries compared to MSC-injected Matrigel plugs (Fig. S5). However, the conditioned medium of MSCs, SVFs, and MVFs exhibited similar protein levels of paracrine factors, such as IL-6, IL-8, IL-10, and VEGF, and pro-angiogenic activities including endothelial cell migration and tube forming ability (Fig. S6). These results suggest that MVFs may have greater vasculogenic potential than SVFs or MSCs through direct incorporation into blood vessels in vivo.

### Therapy of hindlimb ischemia with MVF transplantation

**Fig. 4:**
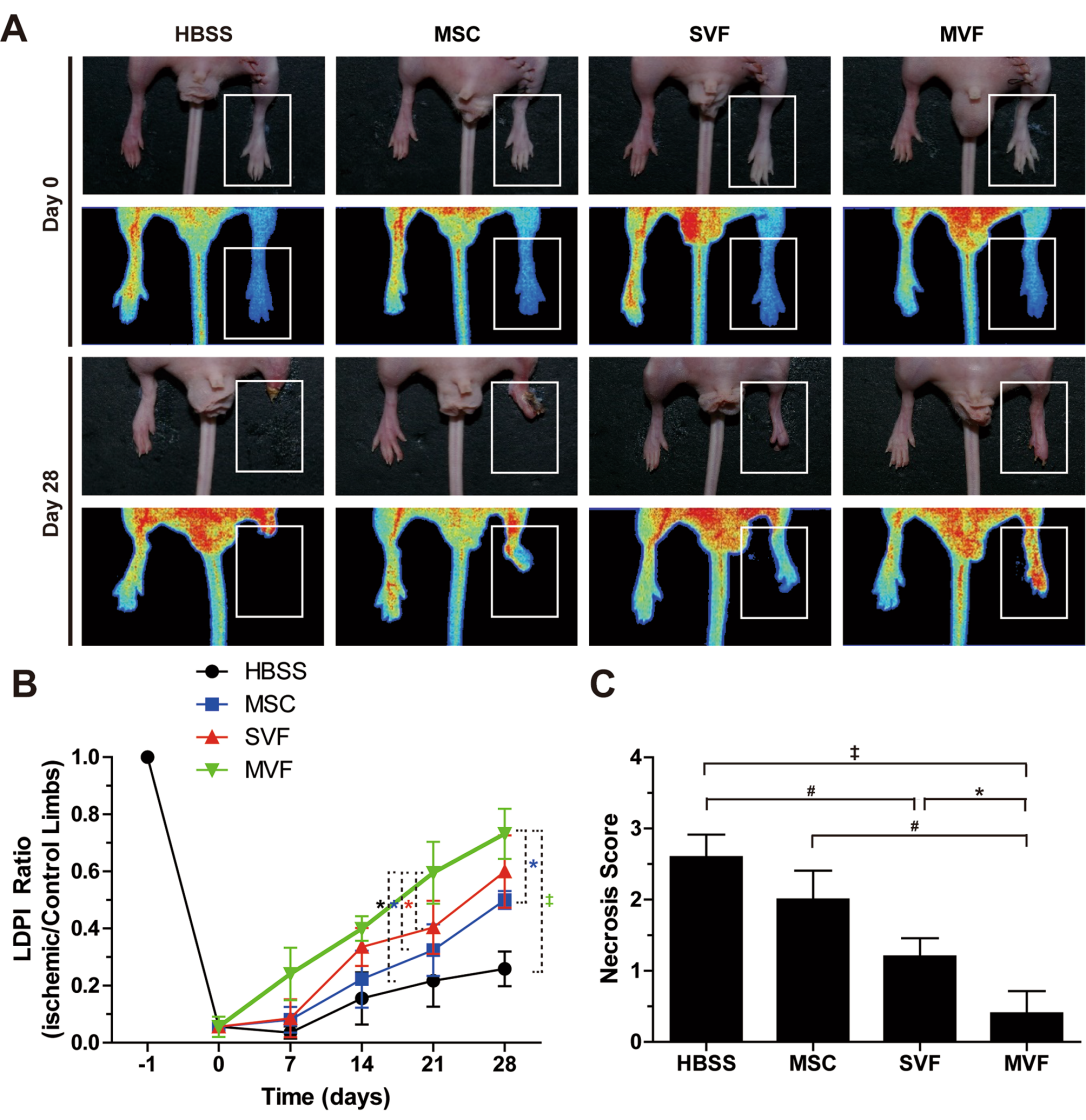
**Effects of MVF, SVF, and MSC transplantation on blood perfusion and tissue necrosis in a murine hindlimb ischemia model** (**A**) Representative photographs and laser Doppler perfusion imaging (LDPI) of mouse hindlimbs on days 0 and 28 after injections of HBSS, MSCs, SVFs, or MVFs. White boxes indicate the regions for LDPI measurement. (**B**) Quantitative analysis of the blood perfusion recovery measured by an LDPI analyzer. Data are presented as mean ± SD (n = 5). (**C**) Statistical analysis of the necrosis score on day 28. Data indicate mean ± SD (n = 5). ^‡^p < 0.005, ^#^p < 0.01, *p < 0.05

To explore the therapeutic efficacy of MVFs, a murine ischemic hindlimb was administered with MVFs, SVFs, or MSCs. To compare the therapeutic efficacy of MSCs, SVFs, and MVFs, the number of cell components in MSCs, SVFs, and MVFs were counted and equal numbers of viable cells (1 × 10^6^ cells/mice) were transplanted into ischemic limbs. Transplantation of MVFs increased blood flow in the ischemic limbs (Fig. 4A and B). The LDPI ratio of hindlimbs transplanted with MVFs was significantly greater than that of hindlimbs transplanted with SVFs or MSCs. The degree of tissue necrosis in MVF-injected ischemic hindlimbs was substantially lower than in MSC- and SVF-injected limbs (Fig. 4C). MSCs, SVFs, and MVFs increased blood flow and alleviated tissue necrosis in the ischemic limbs in a dose-dependent manner, with MVFs having a greater therapeutic effect than MSCs and SVFs (Fig. S7).

### Transplantation of MVFs stimulates neovascularization in ischemic limbs

**Fig. 5:**
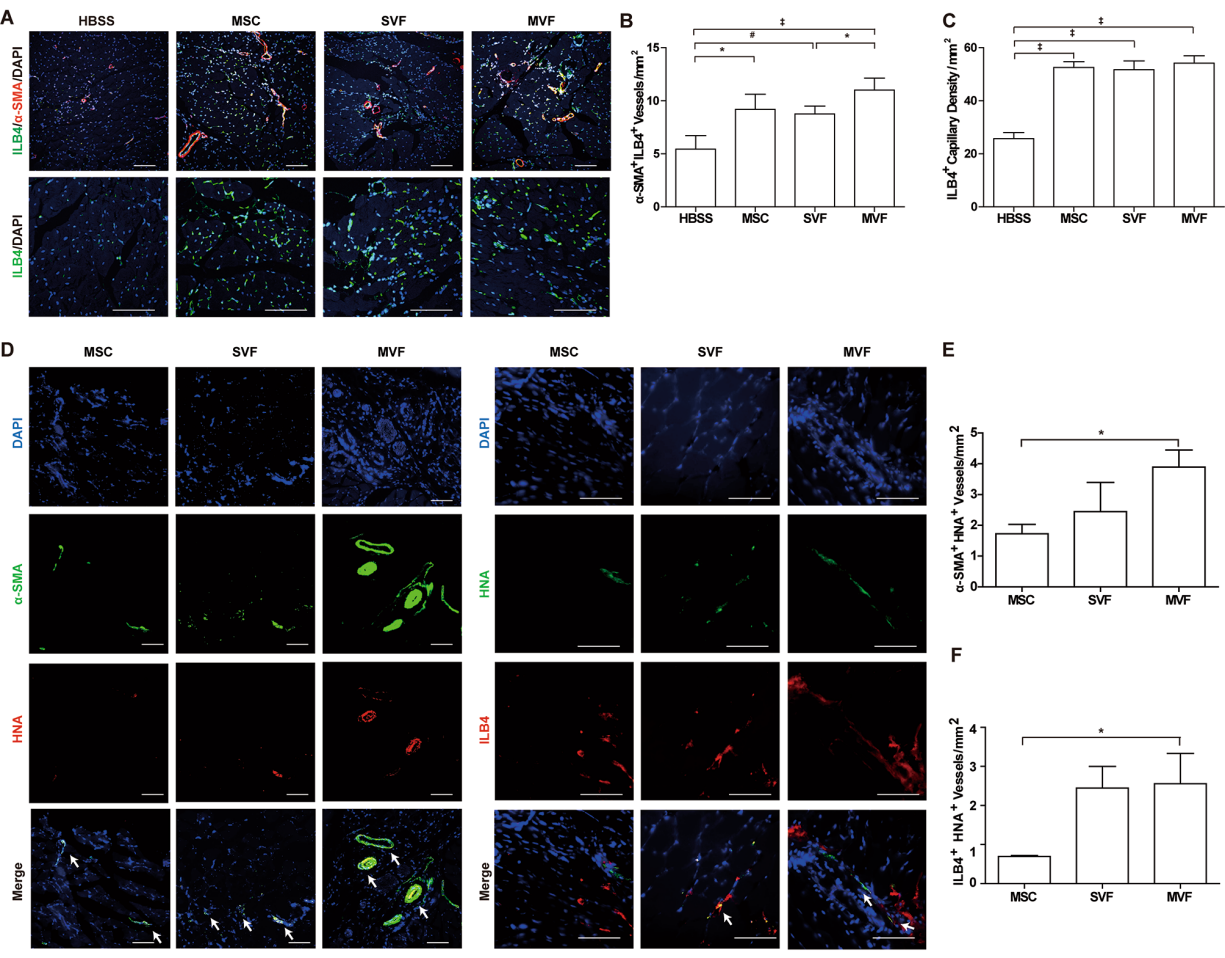
**Effects of MVF, SVF, and MSC transplantation for**in vivo**neovascularization in ischemic hindlimb.** (**A**) Immunostaining of ILB4 capillaries (green) and α-SMA blood vessels (red) with nuclei staining by DAPI (blue) in ischemic limbs injected with HBSS, MSCs, SVFs, or MVFs at 28 days after surgery. (**B**) Quantification of ILB4 and α-SMA double positive arteries in the ischemic limbs by immunostaining (**C**) Quantification of ILB4 positive capillary in the ischemic limbs by immunostaining. (**D**) Representative images of ILB4-positive capillaries and α-SMA-positive blood vessels in ischemic limbs transplanted with MSCs, SVFs, or MVFs at 28 days after surgery. Nuclei were counter-stained with DAPI (blue color). The white arrows indicate the HNA-positive blood vessels. (**E**) Quantification of α-SMA- and HNA-double positive arteries in the ischemic limbs by immunostaining. (**F**) Quantification of ILB4- and HNA-double positive capillaries in the ischemic limbs by immunostaining. Data is presented as mean ± SD (n = 5). Scale bar = 100 μm ^‡^p < 0.005 ^#^p < 0.01, *p < 0.05

To explore the effect of MVFs on vasculogenesis in vivo, ischemic hindlimb muscles were collected at day 28 after transplantation of MVFs and immunostained with anti-α-SMA and isolectin B4 (ILB4) antibodies. The number of α-SMA- and ILB4-double positive blood vessels markedly increased in ischemic limbs transplanted with MSCs, SVFs, and MVFs compared to that in buffer-injected limbs. The number of α-SMA- and ILB4-double positive blood vessels in the MVF-injected limbs was greater than that in the SVF-injected limbs (Fig. 5A and B). Moreover, the number of ILB4-positive capillaries increased in hindlimbs transplanted with MSCs, SVFs, and MVFs compared to that in buffer-injected limbs (Fig. 5A C).

To explore the differentiation of the MVFs transplanted into blood vessels in vivo, ischemic limbs were stained with antibodies against α-SMA and HNA. The number of HNA- and α-SMA-double-positive vessels in MVF-injected limbs was greater than in ischemic limbs injected with SVFs or MSCs (Fig. 5D and E). Moreover, the number of ILB4- and HNA-double-positive vessels increased in limbs injected with SVFs or MVFs (Fig. 5F). To confirm these results, the tissue specimens were stained with a human-specific β-actin antibody together with anti-α-SMA or anti-CD31 antibody. The numbers of human-specific β-actin-positive blood vessels expressing α-SMA or CD31 were significantly increased in MVF-injected limbs compared to MSC- or SVF-treated groups (Fig. S8). Taken together, these results suggest that MVFs has greater vasculogenic potential than SVFs or MSCs.

## Discussion

The SVFs contain cells with a vascular lineage and inflammatory cells, which can cause inflammation in the recipients. In the present study, we modified the isolation protocol for MVFs using collagenase treatment and size-specific sieving using nylon mesh with different pore sizes to enhance the purity of microvascular fractions. First, the MVFs were collected using a 100-µm nylon mesh after filtration with a 500-µm nylon mesh to remove single cells. Second, the nylon mesh was pre-coated with BSA to prevent non-specific adherence and loss of MVFs during filtration. Reportedly, MVF-derived cells have MSC-like multipotent differentiation potential [39]. In the present study, we showed that MVFs and SVFs express several MSC markers, such as CD29, CD44, and CD90. However, CD105 did not express in MVFs and SVFs, suggesting that MVFs and SVFs are phenotypically different from the cultured MSCs. EPCs have been reported to express CD31 and CD34 [40, 41]. We found that MVFs possessed a higher level of CD34-positive cells than SVFs. These results suggest that MVFs have a greater number of CD34-positive EPCs than SVFs. However, MVFs possessed lower levels of F4/80-positive macrophages than SVFs, suggesting that MVFs has a more enriched population of vascular stem and progenitor cells and lymphatic vessel fragments.

Reportedly, MVF-like microvascular tissues stimulate neovascularization by incorporation into transplanted tissues [42]. The transplanted MSCs and SVFs also form blood vessels in vivo [43]; however, the engraftment and neovascularization capabilities of MSCs and SVFs are quite low [44]. In the present study, using a Matrigel plug assay, we demonstrated that MVFs stimulate in vivo angiogenesis. In particular, MVFs administration increased the number of newly formed microvessels with a concomitant increase in hemoglobin content. Increased neovascularization correlated with the incorporation of MVFs into newly formed microvessels. The number of HNA-positive microvessels increased more following the administration of MVFs than by either SVFs or MSCs, suggesting a greater potential of MVFs for neovascularization. In addition, MVFs caused an increase in lymphangiogenesis in a murine lymphedema model [34, 45]. MVFs retain its native structure and cell composition, contributing to the formation of vascularized adipocyte organoids in vitro [46]. MVF-seeded scaffolds exhibit higher vascularization than SVF-seeded scaffolds after in vivo implantation [30]. These results suggest that MVFs has greater in vivo neovascularization potential than SVFs and MSC *via* its increased engraftment efficiency and blood vessel-forming ability. MVFs stimulate cutaneous wound healing by improving the vascularization, lymphangiogenesis, and integration of dermal skin substitutes. Since MSCs, SVFs, and MVFs exhibited similar paracrine and pro-angiogenic activities in vitro (Fig. S6), it is likely that the enhanced engraftment efficiency of MVFs may be responsible for the greater neovasculogenic activities of MVFs in vivo.

Intramuscular injection of autologous MSCs (1 × 10^8^ cells/patients) into the ischemic leg of patients increased trans-cutaneous oxygen pressure and improved wound healing [47]. Moreover, allogenic adipose-derived MSCs (1 ~ 2 × 10^6^ cells/kg of body) have been transplanted into ischemic limbs of diabetic patients with critical limb ischemia [48]. Intramuscular injection of autologous adipose-derived SVFs (19 ~ 157 × 10^6^ cells/patients) has been reported to treat critical limb ischemia in a clinical trial [49]. It is possible to isolate 2 × 10^6^ MVFs from 100 mL lipoaspirates of a donor, and each MVF is consisted of about 10 cells, suggesting that 2 × 10^6^ MVFs corresponds to approximately 2 × 10^7^ cells. Since the LDPI ratio in the ischemic limbs transplanted with 1 × 10^5^ MVFs is higher than that in the ischemic limbs injected with 5 × 10^5^ MVFs (Figure S7), 2 × 10^7^ cells from MVFs are likely equivalent to 1 × 10^8^ MSCs, which is the clinical dose transplanted in patients. Therefore, autologous adipose-derived MVFs isolated from 100 mL lipoaspirates are sufficient for the treatment of patients with critical limb ischemia, proposing usefulness of MVFs as a novel cell therapy. Despite the strong therapeutic efficacy of MVFs, it has been reported that MVFs from aged donor mice exhibited an impaired vascularization [50]. In addition, patients older than 65 years of age are generally considered ineligible for autologous stem cell transplantation. Since the prevalence of critical limb ischemia is highest in elderly patients, and autologous transplantation of MVFs for the treatment of critical limb ischemia may be limited in elderly patients. To improve the therapeutic efficacy of MVFs, allogenic MVFs isolated from healthy young donors may be considered for the treatment of critical limb ischemia. Moreover, MVFs can be cryopreserved without affecting their length distribution and cellular composition [51]. Although the potential of immune rejection upon allogenic transplantation of MVFs remains to be validated, these results suggest that MVFs isolated from healthy donors can be cryopreserved for allogenic transplantation.

## Conclusions

The present study demonstrates for the first time that adipose tissue-derived MVFs are functional vessel segments and highly effective for treating peripheral artery disease owing to their improved engraftment efficiency and neovasculogenic activity. Therefore, MVFs are highly useful for treating ischemic diseases and in tissue engineering containing blood vessels.

Adipose tissue-derived microvascular fragments are vascularization units in regenerative medicine and tissue engineering containing microvascular networks. Intramuscular injection of microvascular fragments markedly increased blood flow in the ischemic hindlimbs and alleviated tissue necrosis. The present study suggests that microvascular fragments show improved engraftment efficiency and vasculogenic activity in vivo and are highly useful for treating ischemic diseases and in tissue engineering.

## Electronic supplementary material

Below is the link to the electronic supplementary material.

Supplementary Material 1

## Data Availability

The data related to this article will be shared upon reasonable request to the corresponding author.

## Abbreviations

PAD: Peripheral artery disease
SVFs: Stromal vascular fractions
MVFs: Microvascular fractions
MSCs: Mesenchymal stem cells
BSA: Bovine serum albumin
EPCs: Endothelial progenitor cells
VEGF: Vascular endothelial growth factor
HNA: Human nuclear antigen
