# Supplementary material for: Transplantation of adipose tissue-derived microvascular fragments promotes therapy of critical limb ischemia
Source: Biomater Res. 2023 Jul 16;27:70. doi: 10.1186/s40824-023-00395-6 (PMC10350266; doi:10.1186/s40824-023-00395-6)
Supplement: Supplementary file 1 — Supplementary Material 1 [file 40824_2023_395_MOESM1_ESM.pdf]

Supplementary Information

Transplantation of Adipose Tissue-Derived Microvascular Fragments Promotes Therapy of Critical Limb Ischemia

Gyu Tae Park[1], Jae Kyung Lim[1], Eun-Bae Choi[1], Mi-Ju Lim[1], Bo-Young Yun[2], Dae Kyoung Kim[1], Jung Won Yoon[1], Yoon Gi Hong[3], Jae Hoon Chang[3], Seong Hwan Bae[4], Jung Yong Ahn[2¶], and Jae Ho Kim[1¶]

[1]Department of Physiology, College of Medicine, Pusan National University, Yangsan 50612, Gyeongsangnam-do, Republic of Korea; [2]UVA Surgery Clinic, Busan 47537, Republic of Korea; [3]BS The Body Aesthetic Plastic Surgery Clinic, Busan 47287, Republic of Korea; [4]Department of Plastic and Reconstructive Surgery, College of Medicine, Pusan National University, Busan 49241, Gyeongsangnam-do, Republic of Korea.

¶**Corresponding Author:** Jae Ho Kim, Ph.D., Department of Physiology, Pusan National University School of Medicine, Yangsan 50612, Gyeongsangnam-do, Republic of Korea. Tel.: 82-51-510-8073, Fax: 82-51-510-8076, E-mail: jhkimst@pusan.ac.kr; Jung Yong Ahn, M.D., Ph.D., [4]UVA Surgery Clinic, Busan 47537, Republic of Korea. Tel: 82-51-868-4141, E-mail: luckysebu2@naver.com

Supplementary Tables

Table S1. List of antibodies used for immunohistochemistry staining and FACS analysis

Table S1

Antibody	Host	Specificity	Vendor	Identifier
APC-CD29	Mouse	Human	BD bioscience	Cat# 559883
APC-CD44	Mouse	Human	BD bioscience	Cat# 559942
APC-CD45	Mouse	Human	BD bioscience	Cat# 560178
FITC-CD31	Mouse	Human	BD bioscience	Cat# 555445
FITC-CD34	Mouse	Human	BD bioscience	Cat# 555824
FITC-CD38	Mouse	Human	BD bioscience	Cat# 555459
PE-Cy™7-CD90	Mouse	Human	BD bioscience	Cat# 561558
PE-CD105	Mouse	Human	R&D systems	Cat# FAB10971P
PE-CD117	Mouse	Human	BD bioscience	Cat# 561682
Anti-human nuclear antigen (HNA, Clone 235-1)	Rabbit	Human	Millipore	Cat# MAB1281
Ulex Europaeus Agglutinin I (UEA I), Rhodamine		Human	Vector Lab.	Cat# RL-1062
Anti-beta-Actin (clone RM112)	Rabbit	Human	Sigma	Cat# MABT523
Anti-Endoglin(CD105)		Human	Santa Cruz	Cat# SC-20072
Anti-CD44	Mouse	Human	Cell Signaling Technology	Cat# 5640S
Anti-vWF	Rabbit	Human	Abcam	Cat# Ab6994
Anti-vimentin	Rat	Human & Mouse	R&D Systems	Cat# MAB2105
Griffonia Simplicifolia lectin I (GSL I) isolectin B4 Biotinylated		Mouse	Vector Lab.	Cat# B-1205-.5
Anti-alpha smooth muscle Actin (α-SMA)	Rabbit	Mouse	Abcam	Cat# Ab5694
Anti-CD31	Rat	Mouse	BD bioscience	Cat# 550274

Table S1. Park, et al

Table S2. Medical Information of the Liposuction Donors

Donor Number	Age	Gender	BMI	Source
1	35	M	26.5	Aspirate (Liposuction)
2	23	F	26.2	Aspirate (Liposuction)
3	23	F	26	Aspirate (Liposuction)
4	32	M	30.4	Aspirate (Liposuction)
5	39	F	24.5	Aspirate (Liposuction)
6	22	M	27	Aspirate (Liposuction)
7	26	F	20.4	Aspirate (Liposuction)
8	39	F	21.5	Aspirate (Liposuction)
9	44	F	21.9	Aspirate (Liposuction)
10	25	F	23.4	Aspirate (Liposuction)
11	44	F	21.9	Aspirate (Liposuction)

Supplementary Figures

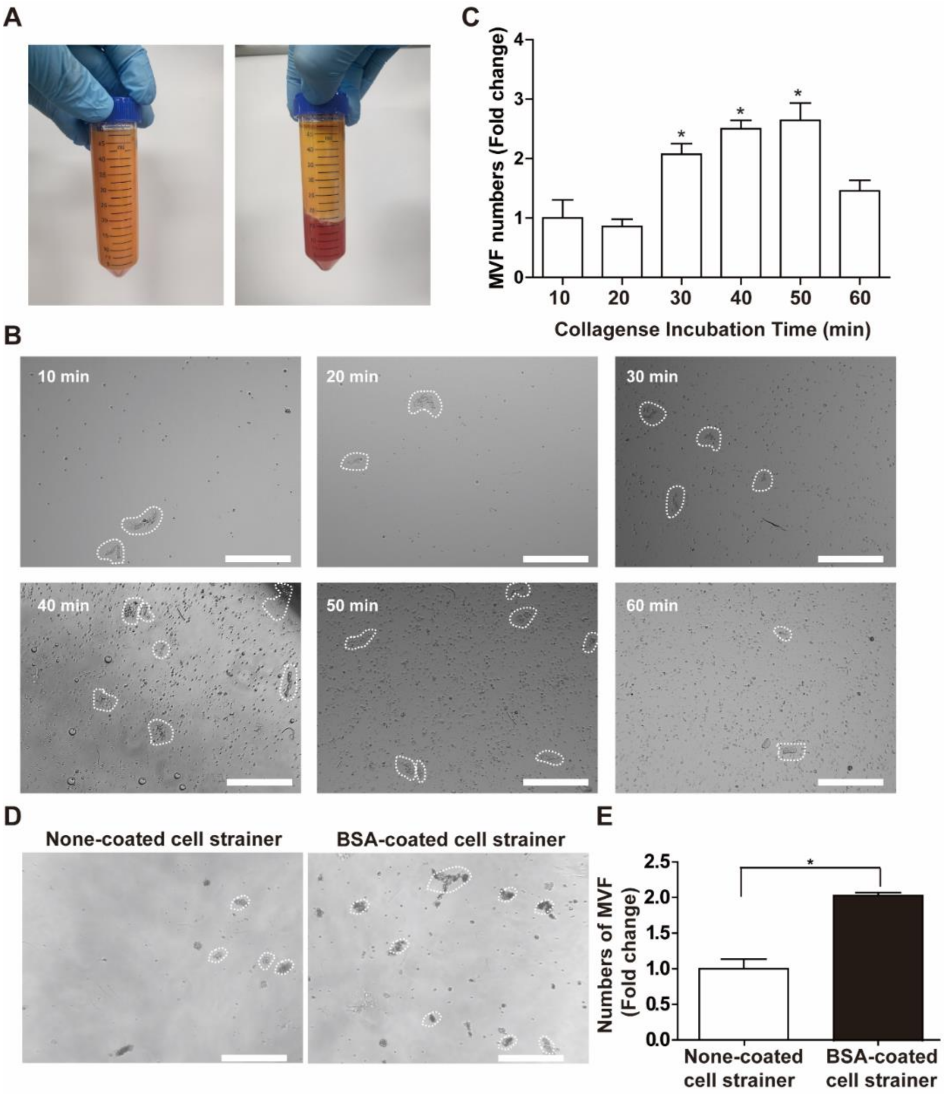

Figure S1. Time-dependent effects of collagenase treatment of human lipoaspirates for isolation of MVF. (A) Adipose tissues were obtained by liposuction at 50ml. (Right) Collected liposuction tissues were centrifuged to reject body fluids, and using supernatant (Left). (B-C) Time-dependent effects of collagenase treatment of human lipoaspirates for isolation of MVF. Image of MVFs isolated from human lipoaspirates. Human lipoaspirates were treated with collagenase for the indicated time periods, followed by serial filtration with Nylon meshes containing 500 µm and 100 µm pores. The MVFs which had been attached on the 100 µm cell strainer were harvested and the photograph images of MVFs were captured (B), and the number of MVFs were analyzed by ImageJ (C) (C-D) Effects of BSA coating of the cell strainer for improved MVF isolation. (D) The collagenase digests of lipoaspirates were sieved through BSA-coated or non-coated nylon meshes. The MVFs purified by using 100 µm nylon meshes were photographed. Image of MVFs isolated from human lipoaspirates. (E) The numbers of MVFs isolated from lipoaspirates were quantified. The lipoaspirates were treated with collagenase for the indicated time periods, followed by quantification of the numbers of MVFs after sieving of MVFs.

Dashed circle line indicates MVF. Scale bar = 400 μm.   Data indicate mean ±SD (n=4). *p<0.05 *vs* 10 min.

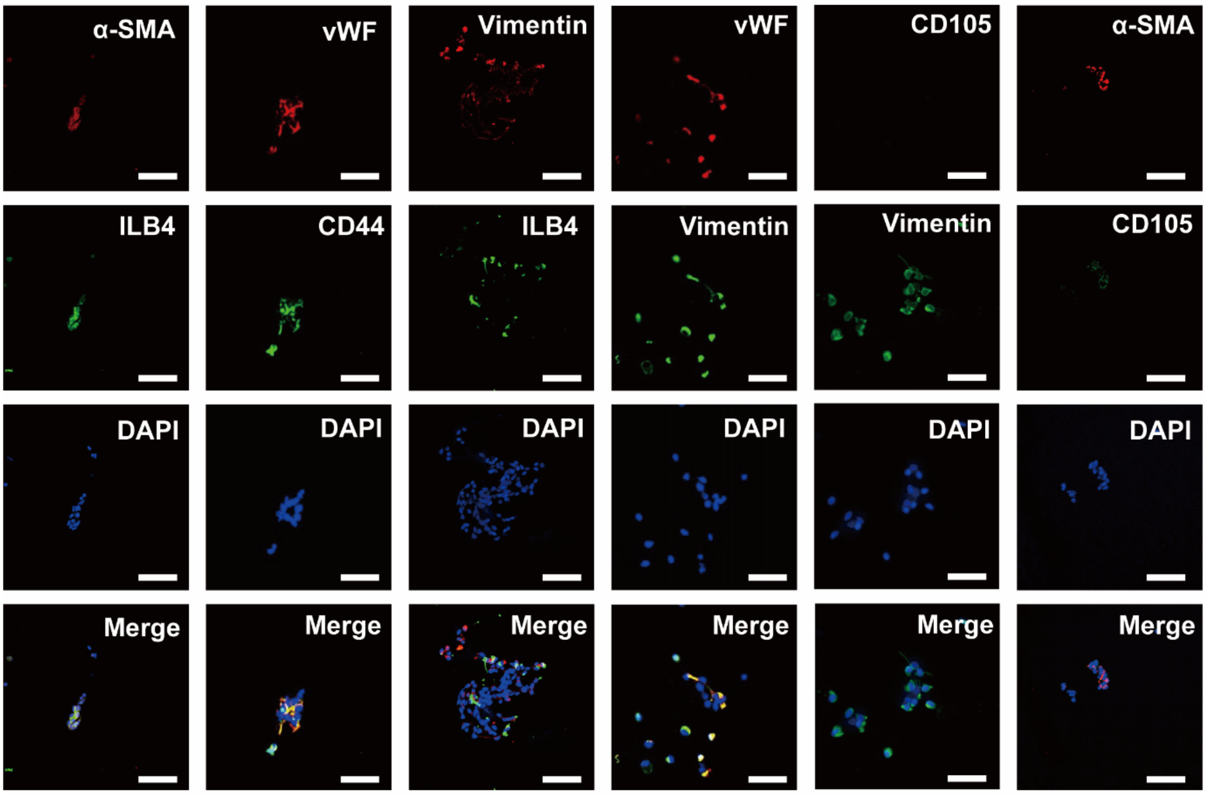

Figure S2. Characterization of microvascular fragments by immunocytochemistry. Immunostaining of MVF using antibodies against vascular endothelial cell markers (ILB4, vWF), smooth vessel markers (α-SMA, Vimentin), and MSC markers (CD44, CD105). Nuclei were stained with DAPI and overlaid images were shown. Scale bar = 100 μm

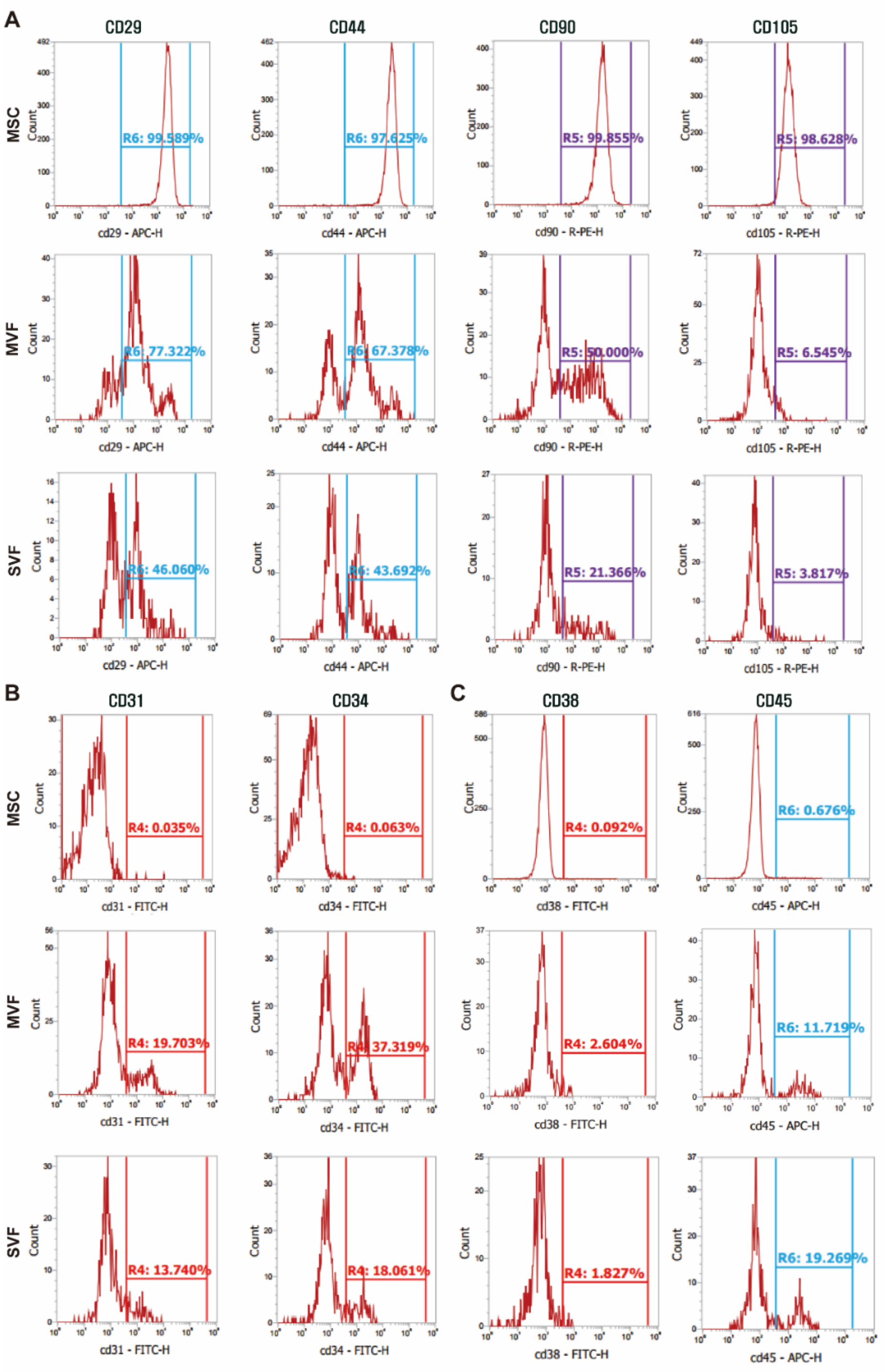

Figure S3. Phenotypic characterization of the MVFs, SVFs, and MSCs isolated from adipose tissues (A) Flow cytometric analysis of MVFs, SVFs, and MSCs with antibodies against MSC markers (CD29, CD44, CD90, and CD105). (B) Flow cytometric analysis of MVFs, SVFs, and MSCs with antibodies against EPC markers (CD31 and CD34). (C) Flow cytometric analysis of MVFs, SVFs, and MSCs with antibodies against immune cell markers (CD38 and CD45). The percentages of the FACS-positive population are indicated.

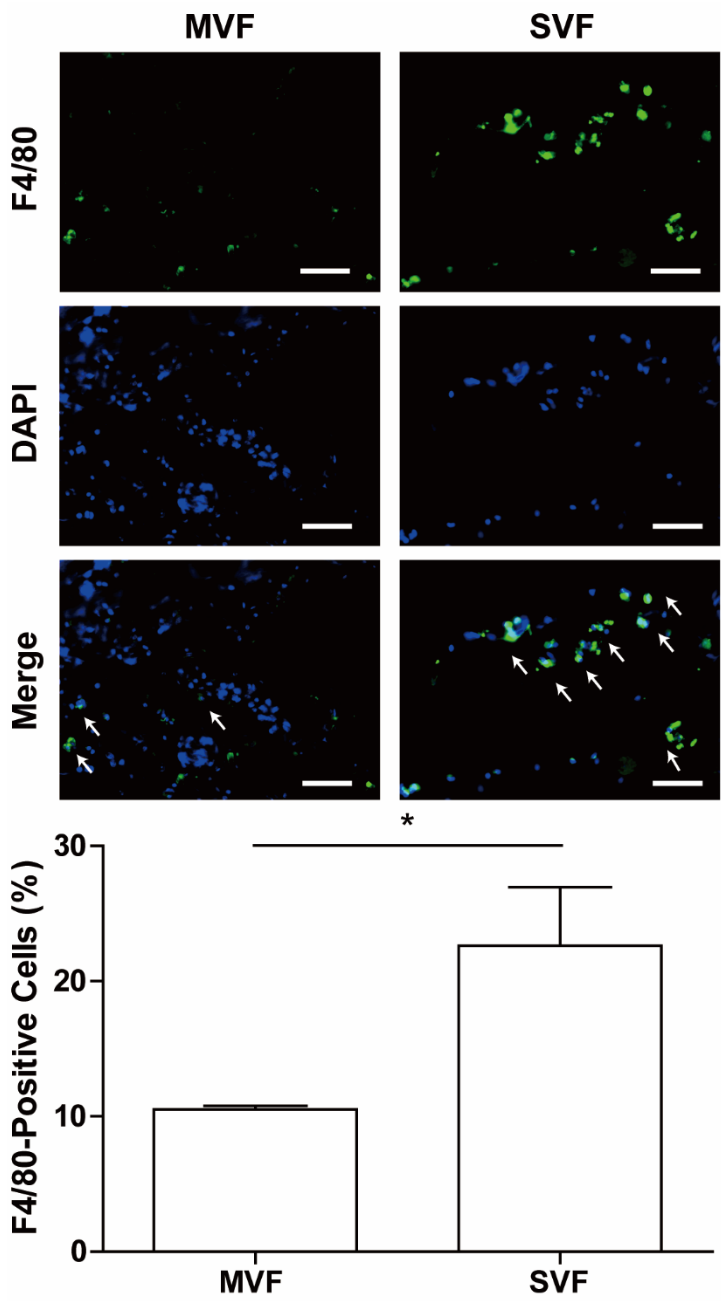

Figure S4. Macrophage levels of microvascular fragments by immunocytochemistry. Immunostaining of MVF and SVF using antibodies against macrophage marker markers (F4/80). Nuclei were stained with DAPI and overlaid images were shown. Scale bar = 100 μm

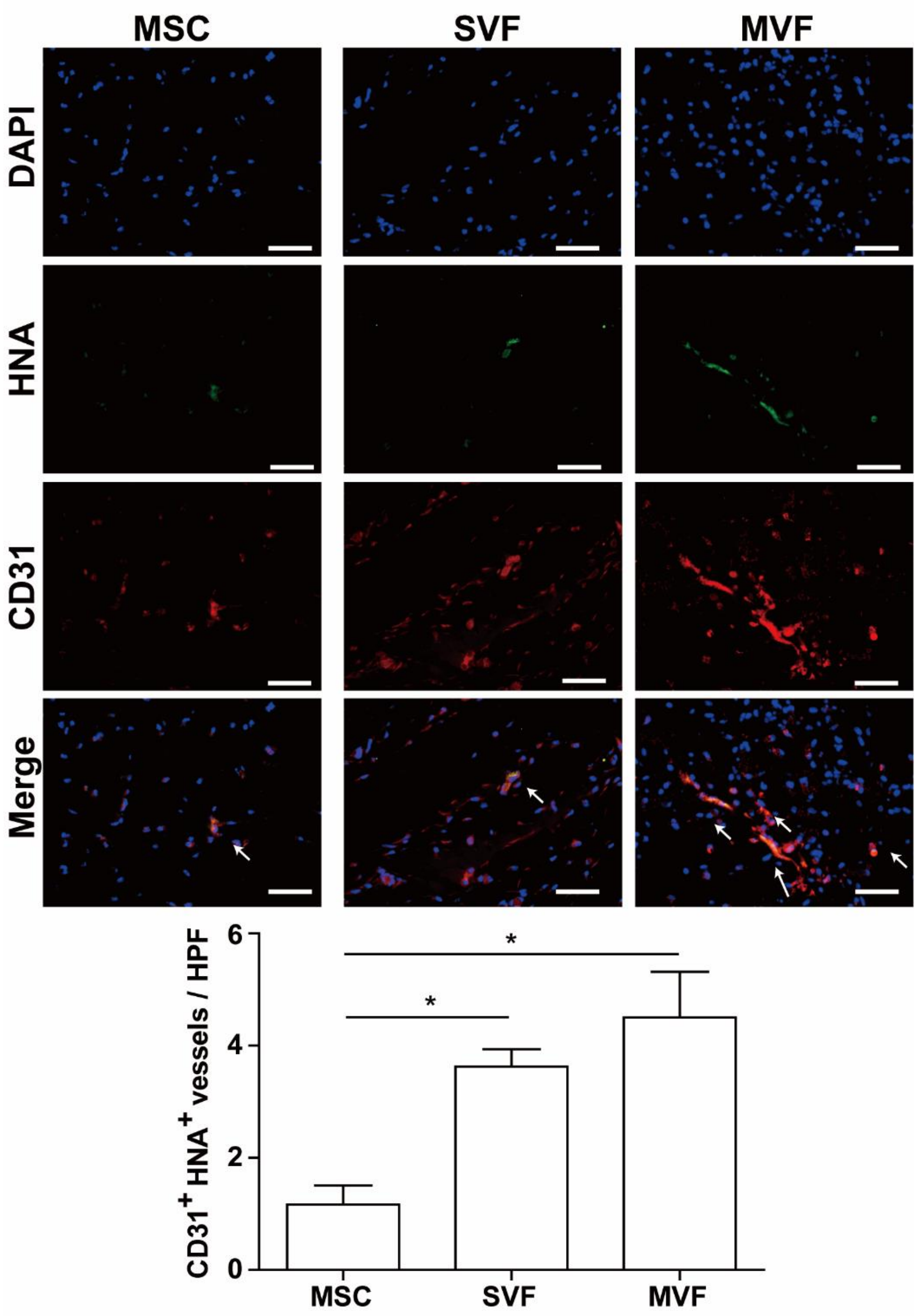

Figure S5. Microvessel formation in Matrigel plugs by MSCs, SVFs, and MVFs. (A) Immunostaining of CD31 capillaries (red) and HNA (green) with nuclei staining by DAPI (blue) in ischemic limbs injected with HBSS, MSCs, SVFs, or MVFs. (B) Quantification of CD31 and HNA double positive microvessels in Matrigel plugs. Scale bar = 100 μm

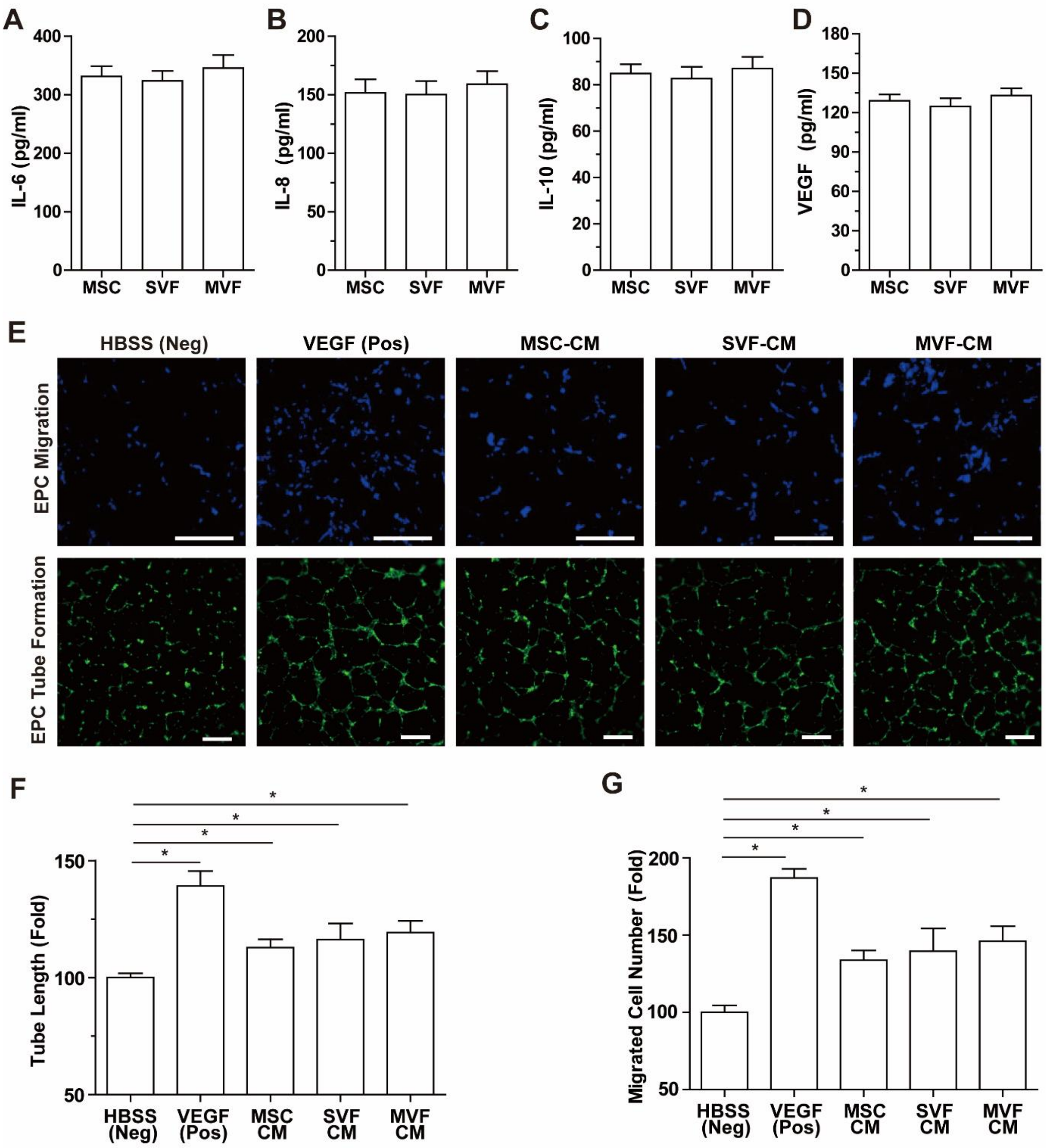

Figure S6. Effects of MSC, SVF, and MVF on migration and tube formation. (A-D) Quantative analysis of cytokines and growth factor by ELISA. Conditioned media were isolated from MSC, SVF and MVF after 48hrs, and analysis of IL-6 (A), IL-8 (B), IL-10 (C) and VEGF (D). IL-6, IL-8, IL-10 and VEGF levels between MSC, SVF, and MVF were not significantly differs in conditioned media. (E) Representative images of chemotactic migration (upper panel) and tube formation (lower panel) of EPC in response to VEGF, MSC, SVF, and MVF-CM are shown. Scale bar = 200 μm. (F) Migration of EPC was measured using a chemotaxis chamber in response to human recombinant VEGF protein (10 ng/ml), MSC-CM, SVF-CM or MVF-CM after a 12- h incubation. (G) EPC was seeded onto a Matrigel-coated dish and treated with VEGF MSC-CM, SVF-CM or MVF-CM for 12 h. Tube formation of EPC was quantified by measuring the length of the tubes formed Data indicate mean ± SD (n=4). *, $p < 0.05$ vs control.

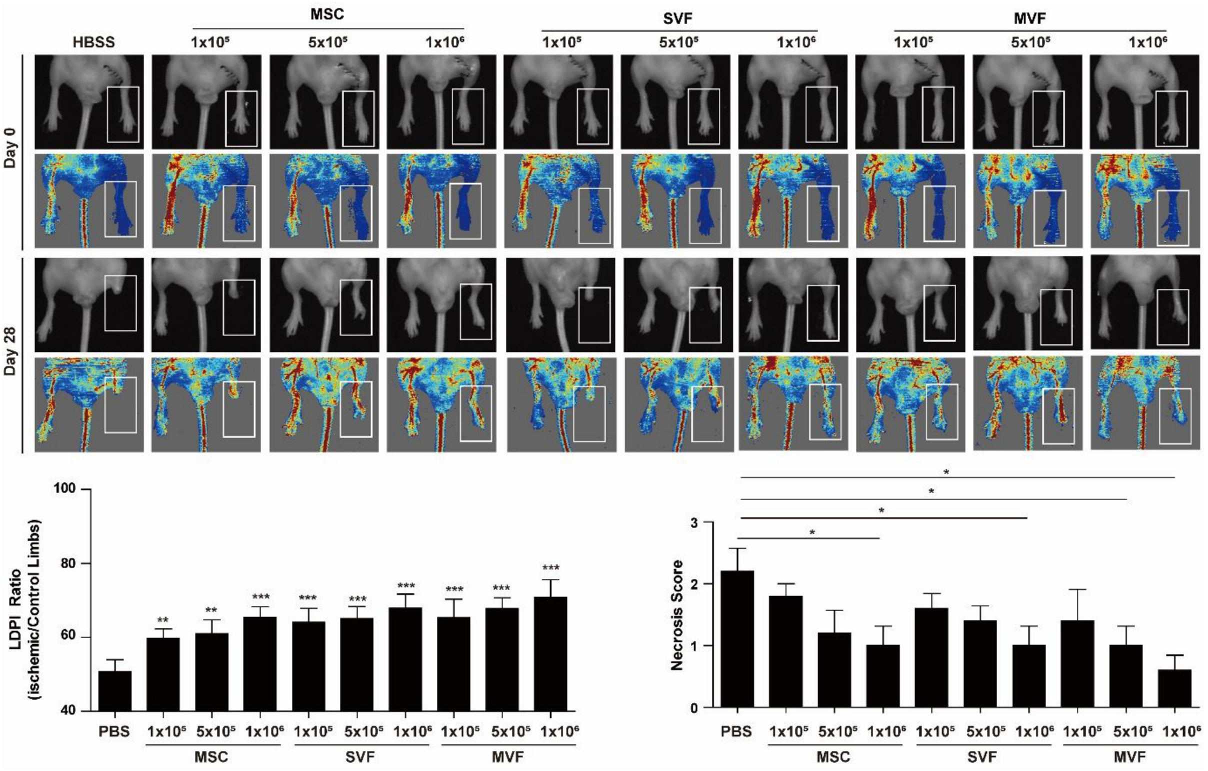

Figure S7. Dose-dependent Effects of MVF, SVF, and MSC transplantation on blood perfusion and tissue necrosis in a murine hindlimb ischemia model (A) Representative photographs and laser Doppler perfusion imaging (LDPI) of mouse hindlimbs on days 0 and 28 after injections of HBSS, MSCs, SVFs, or MVFs. The number of cells injected was 1x10^5, 5x10^5, and 1x10^6 for MSC, SVF, and MVF, respectively. MVF was calculated on a single cell basis. White boxes indicate the regions for LDPI measurement. (B) Quantitative analysis of the blood perfusion recovery measured by an LDPI analyzer. Data are presented as mean ±SD (n=5). (C) Statistical analysis of the necrosis score on day 28. Data indicate mean ±SD (n=5). ‡p<0.005, #p<0.01, *p<0.05

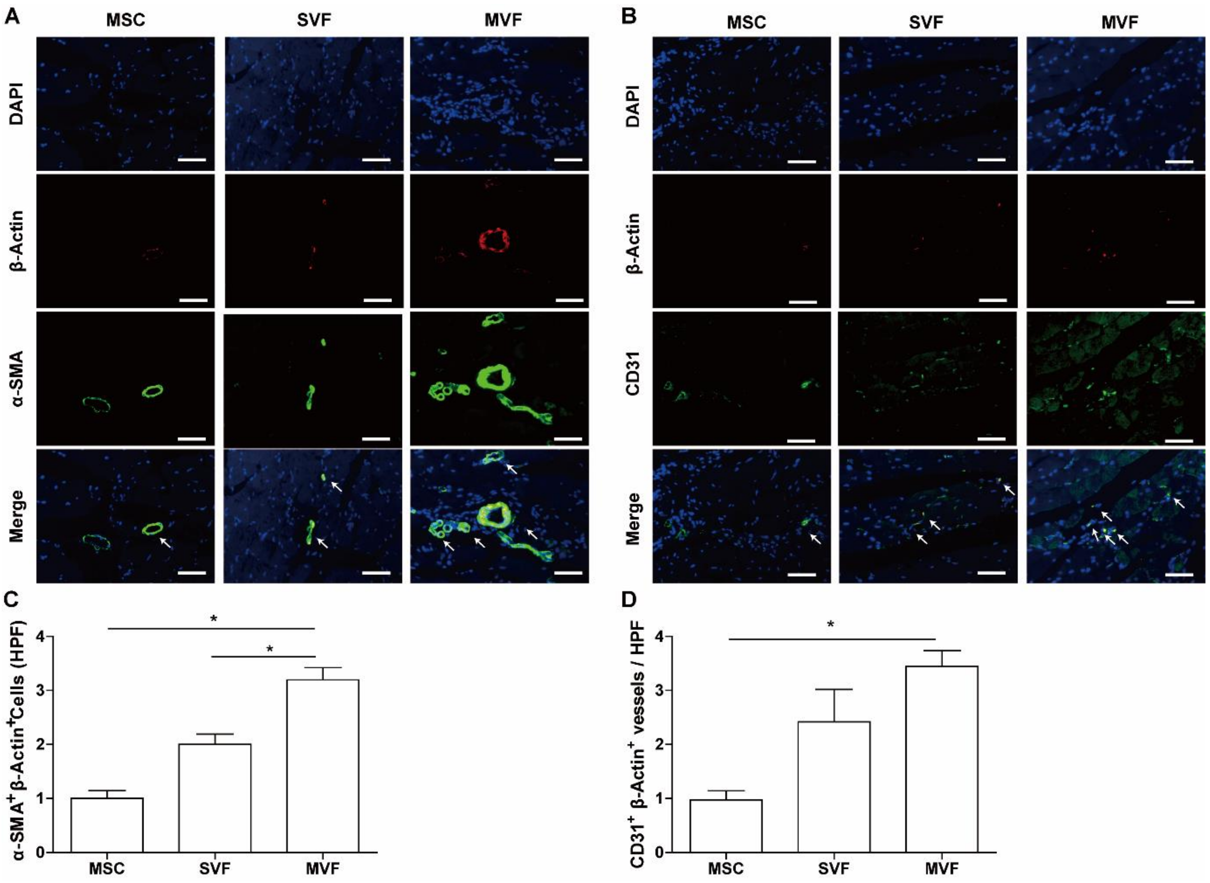

Figure S8. Analysis of MVF, SVF, and MSC transplantation for *in vivo* incorporation into blood vessels formation. (A-B) Representative images of α-SMA-positive vessels, CD31 positive capillaries and β-actin-positive blood vessels in ischemic limbs transplanted with MSCs, SVFs, or MVFs at 28 days after surgery. Nuclei were counter-stained with DAPI (blue color). The white arrows indicate the HNA-positive blood vessels. (C) Quantification of α-SMA- and β-actin-double positive arteries in the ischemic limbs by immunostaining. (D) Quantification of CD31 and β-actin positive capillaries in the ischemic limbs by immunostaining. Data is presented as mean ± SD (n=5). Scale bar = 100 μm ‡p<0.005 #p<0.01, *p<0.05.
